# Preliminary investigation into the identification and management of catatonia in patients admitted to adult inpatient units

**DOI:** 10.1192/bjo.2021.881

**Published:** 2021-06-18

**Authors:** Joanna Moore, Amy Kunicki, Georgina Latcham, Eleanor Perkins, Emma Vaccari

**Affiliations:** 1 Dorset Healthcare University NHS Foundation Trust; 2 Southern Health NHS Foundation Trust

## Abstract

**Aims:**

The prevalence of catatonia is considered to be approximately 10% in psychiatric inpatients. Clinical experience suggests a lower documented prevalence. This could cause longer admissions and complications, such as Neuroleptic Malignant Syndrome (NMS). We carried out a service evaluation to investigate the recognition and management of catatonia on inpatient units in Southern Health Foundation Trust (SHFT). We reviewed the local documented prevalence of catatonia, treatment offered and prevalence of complications.

**Method:**

We retrospectively reviewed the electronic records of 95 consecutive admissions to four adult inpatient units in SHFT, starting on 1st August 2020. We reviewed notes for the admission to establish whether catatonia was suspected and identified. We applied the screening questions from the Bush-Francis Catatonia Rating Scale (BFCRS) to the documented mental state examinations (MSE) prior to, and shortly after, admission. We also recorded the prescriptions issued during the first 72 hours of admission, and whether patients developed neuroleptic malignant syndrome (NMS), serotonin syndrome or required admission to a general hospital during admission.

**Result:**

Catatonia was documented as a possibility for 2 patients (2.1%). One showed possible posturing and stupor, while there were no documented symptoms for the other. In both cases the possibility was discounted by the clinical team. Twelve patients (12.6%) showed one or more possible or confirmed signs of catatonia. Eleven of these were prescribed regular antipsychotic medication on admission, but only 3 were prescribed regular benzodiazepines. NMS was more likely to be suspected in patients with a BFCRS of 1 or more compared with those with a score of 0, with an odds ratio of 8.1 (95% CI [1.03-64.0], Fisher's exact test = 7.79, p = .076).

**Conclusion:**

Catatonia is likely under-recognised and under-treated locally among psychiatric inpatients. Although only approaching statistical significance, the higher rate of suspected NMS in patients showing possible catatonia is noteworthy and needs further investigation. Regular benzodiazepines were not frequently prescribed in this group, while antipsychotics, prescribed in all of these patients, can precipitate NMS. Alternatively, this finding could reflect the overlap in clinical presentation between NMS and catatonia. Data collection was limited by the frequent use of “remote clerking”, in the context of the COVID-19 pandemic. Additionally, the quality of mental state examinations was often not sufficient to draw any conclusions on the possible presence or absence of catatonic symptoms. This project has highlighted practice in need of improvement, which will be further prospectively investigated and improved via a Quality Improvement Project.

